# Case Report: Hepatic Adenoma in a Child With a Congenital Extrahepatic Portosystemic Shunt

**DOI:** 10.3389/fped.2020.00501

**Published:** 2020-08-25

**Authors:** Hannah Glonnegger, Maren Schulze, Simone Kathemann, Sebastian Berg, Hannah Füllgraf, Andrea Tannapfel, Patrick Gerner, Jochen Grohmann, Charlotte Niemeyer, Simone Hettmer

**Affiliations:** ^1^Division of Pediatric Hematology and Oncology, Department of Pediatrics and Adolescent Medicine, Faculty of Medicine, Medical Center-University of Freiburg, University of Freiburg, Freiburg im Breisgau, Germany; ^2^Department of Transplant and General Surgery, University Hospital Essen, Essen, Germany; ^3^Division of Pediatric Radiology, Department of Radiology, Faculty of Medicine, Medical Center-University of Freiburg, University of Freiburg, Freiburg im Breisgau, Germany; ^4^Department of Pathology, Faculty of Medicine, Medical Center-University of Freiburg, University of Freiburg, Freiburg im Breisgau, Germany; ^5^Faculty of Medicine, Medical Center, Institute for Pathology, Ruhr-University Bochum, Bochum, Germany; ^6^Department of Pediatrics and Adolescent Medicine, Faculty of Medicine, Medical Center-University of Freiburg, University of Freiburg, Freiburg im Breisgau, Germany; ^7^Department of Congenital Heart Defects and Pediatric Cardiology, Faculty of Medicine, University Heart Center Freiburg-Bad Krozingen, University of Freiburg, Freiburg im Breisgau, Germany

**Keywords:** congenital extrahepatic portosystemic shunt, hepatic adenoma, child, CEPS, Abernethy malformation

## Abstract

Congenital extrahepatic portosystemic shunts (CEPS), previously also described as Abernethy malformations, are rare malformations in which the extrahepatic portal system directly communicates with the vena cava inferior, thereby bypassing the liver. A hypoplastic portal vein (PV) exists in most cases. CEPS have been associated with the development of liver nodules, ranging from mostly focal nodular hyperplasia (FNH) to hepatic adenoma (HA) and even hepatocellular carcinoma (HCC). Tumor development in CEPS may be due to changes in perfusion pressures, oxygen supply or endocrine imbalances. It is important to rule out CEPS in children with liver tumors, because resection could impede future shunt occlusion procedures, and benign masses may regress after shunt occlusion. Here, we review the case of a 9-years-old male with CEPS and hepatic nuclear Factor 1-alpha (HNF-1-alpha) inactivated HA to raise awareness of this condition and review histopathological changes in the liver of CEPS.

## Introduction

**Figure 1:**
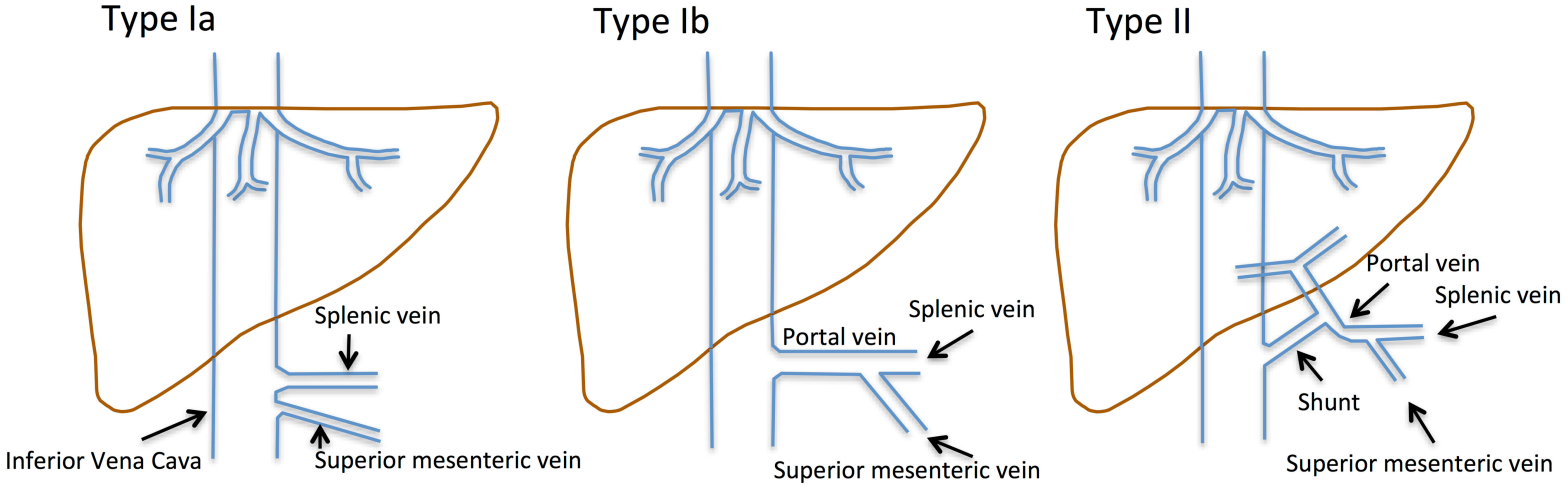
Historical classification of CEPS according to Abernethy and the Morgan and Superina scheme. Type I CEPS was defined as an end-to-end communication of the portal system and the IVC, including type Ia (SMV and SV draining separately into the IVC) and type Ib (SMV and SV join to form a common vessels that feeds into the IVC). Type II CEPS was defined as an end-to-side communication of the PV with the IVC.

Congenital extrahepatic portosystemic shunts (CEPS), previously known as Abernethy malformations, are rare conditions defined by diversion of blood from the splanchnic region away from the liver (Figure 1): Venous blood from the superior mesenteric vein (SMV) and the splenic vein (SV) does not drain into the portal vein (PV), but shunts partially into the inferior vena cava (IVC). A hypoplastic PV is present in most patients.

CEPS classification has evolved over time. Historically, type I CEPS was characterized by an end-to-end communication of the portal system and the IVC (Table 1). Type Ia CEPS with separate drainage of the SMV and SV into the IVC was distinguished from type Ib CEPS, which was defined by merging of the SMV and SV and drainage of a common vessel into the IVC. Type II CEPS was described as an end-to-side communication of the PV and the IVC. More recently, CEPS was reclassified to correlate with clinical significance and therapeutic implications (1–3). For example, Kanazawa et al. (4) suggested classification based upon visualization of the intrahepatic portal system during shunt occlusion tests: Firstly, in mild CEPS, the intrahepatic portal system is seen well into the periphery. Secondly, in moderate CEPS, the intrahepatic portal system exhibits some distribution into the periphery. Thirdly, in severe CEPS, the intrahepatic portal system is seen poorly or not visualized at all.

**Table 1:** Classification of CEPS according to the Morgan and Superina scheme.

**Type**	**Shunt anatomy**
I	End-to-end communication from the portal system to the inferior vena cava.
Ia	Splenic vein and superior mesenteric vein drain separately into the systemic circulation.
Ib	Splenic vein and superior mesenteric vein join to form a common trunk and directly drain into the systemic circulation.
II	Side-to-side anastomosis of the portal vein with the inferior vena cava.

Measurement of portal venous pressures during shunt occlusion should inform clinical decision-making. Portal venous pressure below 25 mm Hg allows for direct shunt occlusion. If portal venous pressure is above 25 mm Hg, the shunt should be occluded as part of a two-step approach (4). It is important to note that portal flow can be restored by permanent shunt occlusion, even if the PV is extremely hypoplastic (5). Baiges et al. demonstrated that 8 out of 39 adult patients with CEPS and lack of a detectable PV exhibited intrahepatic venous flow during a shunt occlusion test (6). This was likely due to a hypoplastic portal vein, which had not been visualized because of inefficient perfusion (7).

CEPS are rare diseases in need of further attention and systematic review. To our best knowledge, ~300 cases have been described in the literature so far. A recent study (6) reported 66 patients with CEPS with a focus on associated health issues; 19 of 66 (29%) patients presented with hepatic encephalopathy, 8 (15%) suffered from pulmonary arterial hypertension, 2 (3%) had hepatopulmonary syndrome, 8 (12%) were diagnosed with hepatocellular carcinoma (HCC) and 10 (15%) with HA. Shunt closure resulted in either resolution or decline of shunt-related problems in 15 of 66 (22%) patients. The shunt was closed in one patient with CEPS and HA, but no subsequent changes in the size of the HA were reported. Similar complications of CEPS were reported by other studies (5, 8, 9).

Here, we report on a 9 years-old boy with HA in the context of CEPS and discuss possible pathomechanisms, that may account for development of liver tumors in this condition.

## Case Description

**Figure 2:**
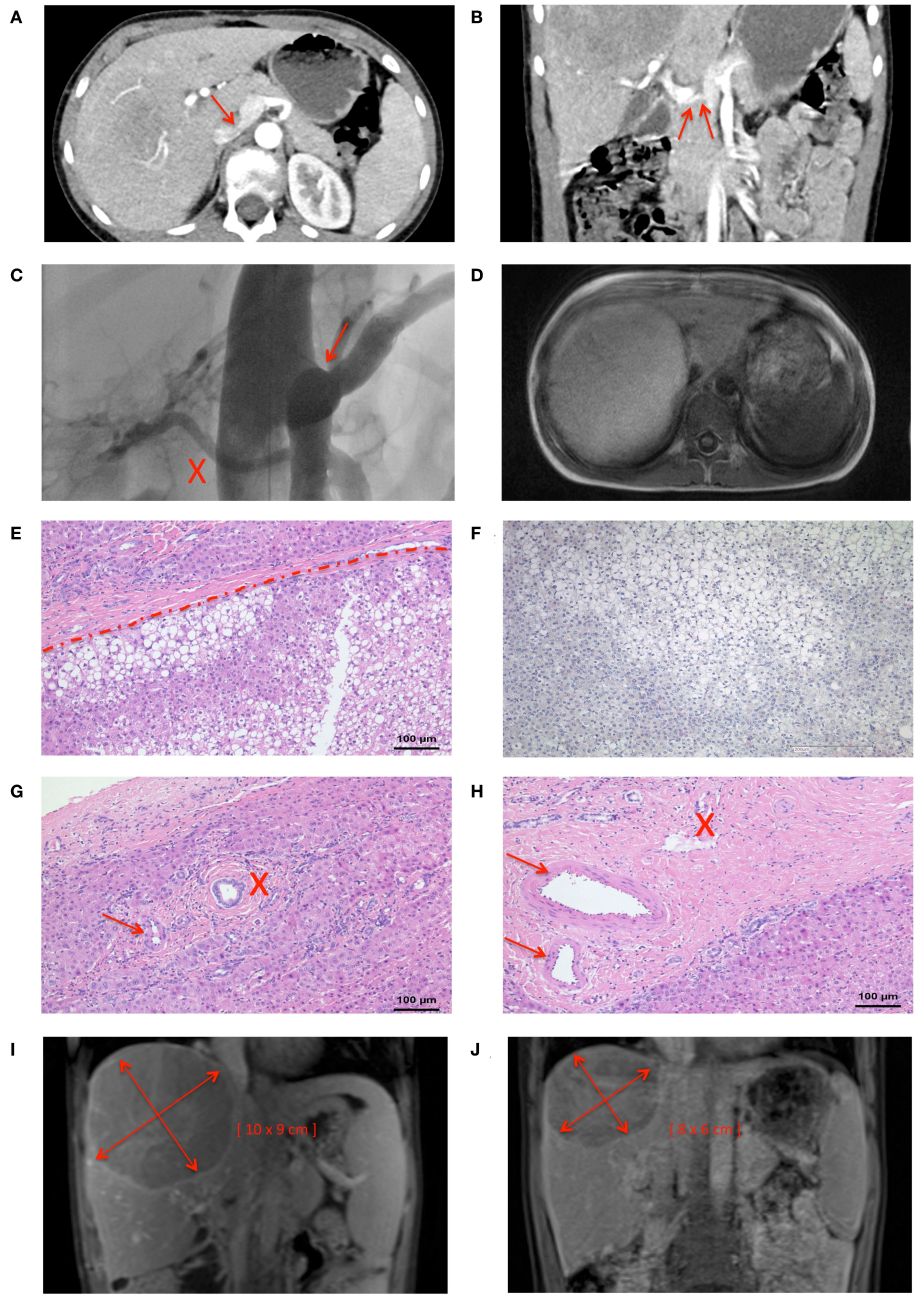
Nine years-old male with Abernethy type II portosystemic shunt and hepatic adenoma. CT-angiography demonstrated **(A)** confluens of the SV, SMV, and IVC (arrow), and **(B)** a hypoplastic PV (arrow). **(C)** The shunt (marked by an arrow) and a hypoplastic portal vein (marked by an X) were clearly visualized during a shunt occlusion test (see also Videos 1, 2). **(D)** MRI imaging confirmed the presence of a large tumor in the liver. **(E)** Histology was consistent with HA with steatosis (lower part), surrounded by normal liver tissue (upper part). **(F)** Negative L-FABP staining in HA tissue indicated HNF1-alpha inactivation. Portal tracts contained **(G)** unpaired arterioles (maked by an arrow) and one bile duct (marked by an X), or **(H)** two arteriolas (marked by an arrow) and one venule (marked by an X). The size of the HA decreased from **(I)** a maximum diameter of 11.5 cm at the time of diagnosis to **(J)** a maximum diameter of 10 cm 4 months after shunt closure.

A 9-years-old male presented with frequent, urgent urination. Ultrasound examination revealed a large tumor (11.5 × 10 × 9 cm) of the right lobe of the liver. The patient reported abdominal pain, headache, pruritus, and a decline in school performance. There was no family history of liver tumors. Magnetic resonance imaging (MRI) of the abdomen revealed fatty remodeling of the tumor in liver segment VII and an extrahepatic portosystemic shunt. Computer tomography (CT)-angiography identified a CEPS type II portosystemic shunt with a hypoplastic PV (Figures 2A,B). Conventional angiography confirmed the presence of a short, patent shunt and a hypoplastic PV, which was visible without full shunt occlusion (Figure 2C, Videos 1, 2). Figure 2D shows size of the adenoma within the right hepatic lobe. Liver function tests, cholestasis parameters, ammonium levels and tumor markers alpha fetoprotein and ß human chorionic gonadotropin were within normal range.

Biopsy of the tumor showed steatosis in ~80% of hepatocytes. Relevant ductal proliferation was absent (Figure 2E). Portal tracts contained unpaired arterioles and one bile duct, or two arterioles and one venule (Figures 2G,H). The neoplastic cell population was negative for glutamine synthetase and glypican-3, both markers of HCC. Likewise, nuclear beta-Catenin expression was absent. Significant fatty changes within the lesion correlated with inactivation of hepatocyte nuclear factor 1 homeobox A. This was accompanied by loss of liver fatty acid binding protein (L-FABP) (Figure 2F) in neoplastic cells, while L-FABP expression was preserved in the surrounding non-neoplastic liver parenchyma. Histological findings were consistent with a diagnosis of hepatic-nuclear factor-1-alpha (HNF-1-alpha) inactivated HA.

Because of the size of the tumor, the CEPS and the presence of a small, rather wide shunt, it was decided to pursue surgical shunt closure. First, the shunt was occluded with a surgical clamp, resulting in a rise in portal venous pressures above 35 mm Hg and visible bowel congestion. The shunt was only partially closed. Four days later, the shunt was closed completely as part of a second procedure, during which portal venous pressure was 22 mm Hg, and no signs of intestinal congestion were noted. The patient received anticoagulation with unfractionated Heparin for 3 days, followed by prophylactic doses of low molecular weight heparin. Five days after the second procedure, anticoagulation was stopped because of concerns for subcapsular bleeding in the liver.

**Figure 3:**
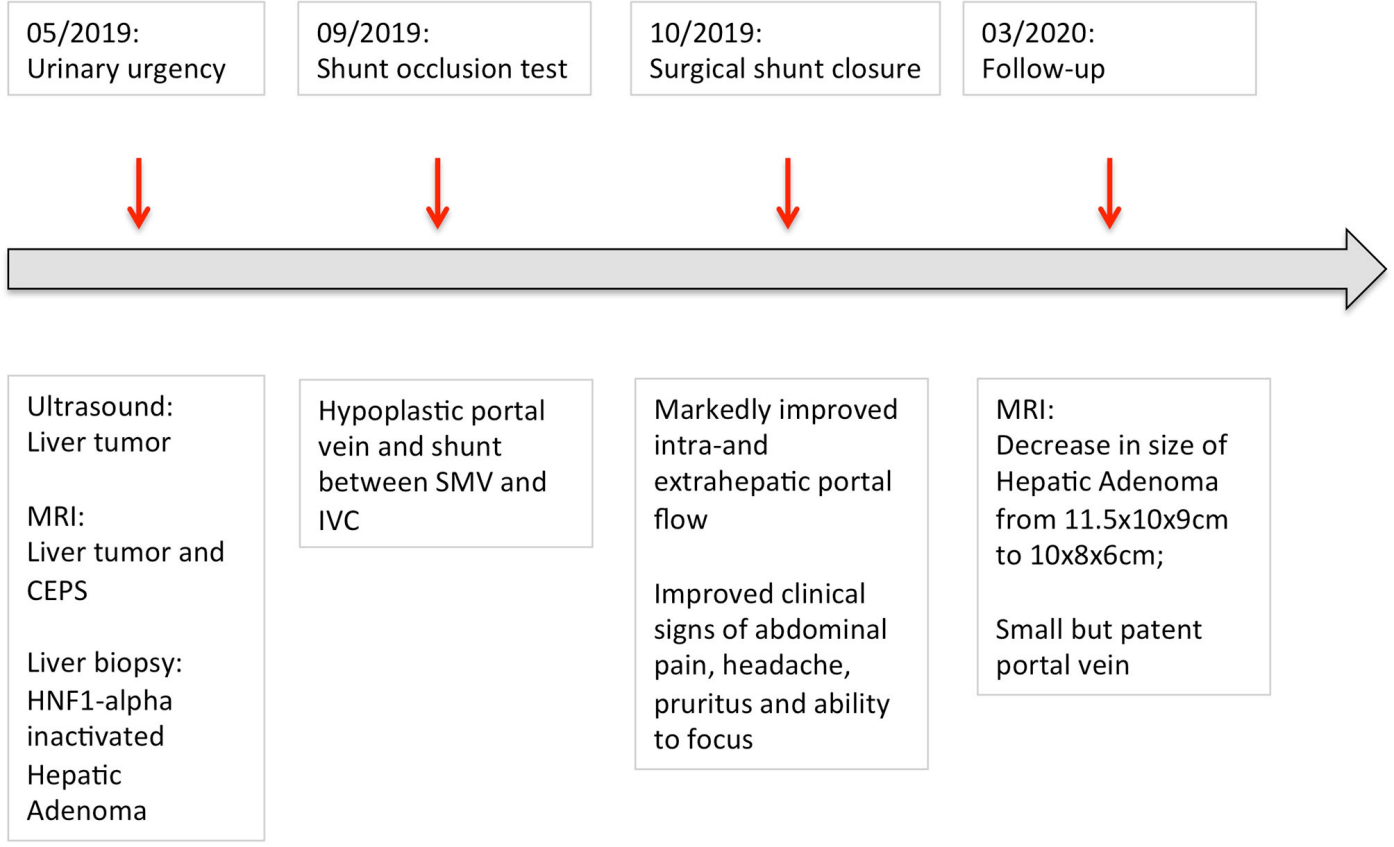
Graphical depiction of the patient's disease course. The patient first presented with urinary urgency in May 2019. Surgical closure of the shunt was performed in October 2019. By March 2019, the size of the adenoma decreased from initially 11.5 × 10 × 9 cm to 10 × 8 × 6 cm.

Follow-up ultrasound demonstrated markedly improved intra- and extrahepatic portal venous flow. The patient's abdominal pain, headache and the pruritus resolved, and his ability to focus in school improved. Four months after shunt closure, MRI imaging demonstrated a small, patent PV and, notably, a marked decrease in the size of the HA (10 × 8 × 6 cm compared to initial diameters of 11.5 × 10 × 9 cm) (Figures 2I,J, 3).

Informed consent was obtained prior to all procedures and for publication of data. Ethical standards in patient care were followed.

## Discussion

HA in children is rare; it accounts for 3.8% of liver tumors diagnosed in children and adolescents aged 0–20 years and FNH accounts for 10.1% (10). Histologically, HAs are characterized by proliferation or hypertrophy of hepatocytes, often associated with remarkable steatosis (11). HAs are generally classified into three different subtypes (12): (1) adenomas with mutations in the tumor suppressor *HNF1-alpha* (≈45%) (9), (2) adenomas with mutations in ß*-catenin* (≈10%) and (3) adenomas with inflammatory features and no specific mutations (≈40%). In patients with CEPS, liver nodules are typically associated with rarefication of venous vessels within the portal tracts (6, 13, 14), hypertrophy of hepatic arterial branches and remodeling of liver architecture (15–17). Progression of HA into HCC has been described in patients with CEPS, including occasional children (18).

The pathogenesis of liver nodules in CEPS continues to be a matter of debate. Oxygen supply appears to be an important driver of tumor development in the liver (19). Several groups suggested that tumor development in CEPS was caused by excessive arterialization and increased oxygen delivery due to perfusion with mostly arterial instead of portal venous blood (15, 20–25). This is supported by the observation that CEPS-associated liver nodules typically arise in liver tissue, which is supplied with predominantly arterial blood due to CEPS type l malformations (6, 21, 22). Also, excessive capillarization appears to be more common in the liver of children with CEPS and coexisting liver masses (17). Lack of portal flow may contribute to enlarging arterial branches, hypertrophy of hepatocytes, and eventually, liver tumors.

Abnormal blood flow to the liver in CEPS is also likely to cause substantial changes in liver perfusion pressures. Previous observations suggest that liver nodules tend to form in between two neighboring liver areas that receive different volumes of blood (9, 26). Also, higher prevalence of liver nodules in patients, who underwent Fontane procedures (27, 28) or suffer from idiopathic portal hypertension (29), were previously linked to higher perfusion pressures and/or higher rates of liver cirrhosis.

The tumor in the patient reported here was characterized by a fatty phenotype and absence of L-FABP staining, consistent with HNF1-alpha inactivation. Published studies examined liver histology in HNF1-alpha knock-out mice and reported striking hepatomegaly, fatty liver disease, central lobular hypertrophy with degeneration of normal hepatocytes and HCC development in animals with homozygous HNF1-alpha depletion (30–32). Biallelic inactivating nonsense and frameshift mutations of the HNF1-alpha gene on chromosome 12q24.2 were detected in 35–50% of HNF1-alpha inactivated HA (12, 33, 34). Heterozygous HNF1-alpha germline mutations were linked to a predisposition to both diabetes and liver adenomatosis (35, 36). As HNF1-alpha inactivation results in elevated rates of fatty acid synthesis, it is not surprising that HNF1-alpha inactivated HA typically exhibit a fatty phenotype and absence of L-FABP staining, which encodes liver fatty acid binding Protein 1 (35). Neither the proportion of CEPS-associated HAs with changes in the expression of HNF1-alpha, nor possible mechanisms of HNF1-alpha inactivation as a consequence of changes in perfusion pressures or oxygen tension are known. Marten et al. reported decreased functional activity of HNF1-alpha transcription factor activity in amino acid-starve rat hepatoma cells (37). In contrast, Mazure et al. demonstrated that severe hypoxia resulted in downregulation of HNF-4, but not HNF1-alpha expression in hepatoma cells (32).

Finally, hormonal imbalances have been discussed as contributing factors in the development of benign liver nodules in patients with CEPS. Diversion of splanchnic blood away from the liver may result in abnormal composition of hepatotrophic substances, including insulin (38) and estrogen (39). However, insulin is being diverted from the liver in patients with CEPS (16). Estrogen, on the other hand side, has been clearly linked to the development of FNH and HA. The annual incidence of HA in women using estrogen-containing contraceptives ranges from 30 to 40 cases per million, as compared to 1–1.3 cases per million among non-users. Discontinuation those contraceptives has been associated with regression of liver tumors (39). Further studies are needed to examine whether prolonged estrogen exposure due to low vascular flow might put patients with CEPS at risk for HA, and whether or not this is associated with HNF1-alpha inactivation.

## Conclusions

CEPS confers a striking susceptibility to the formation of liver nodules. It is likely that changes in oxygen tension, perfusion pressures or supply with hepatotrophic factors contribute to tumor development, although the precise pathogenic mechanisms remain unknown. Full or partial regression of nodules has been observed in patients with CEPS after shunt closure and normalization of blood flow, oxygen supply and exposure to hepatotrophic substances (5, 40). We would like to stress that the diagnosis of a liver tumor in any child warrants careful examination of the portal venous system to identify CEPS and plan treatment accordingly. If vascular malformations are detected, it is extremely important to evaluate treatment of the underlying pathology (CEPS) prior to resection of residual liver masses.

## Data Availability Statement

The original contributions presented in the study are included in the article/Supplementary Material, further inquiries can be directed to the corresponding author/s.

## Ethics Statement

Written informed consent was obtained from the minor(s)' legal guardian/next of kin for the publication of any potentially identifiable images or data included in this article.

## Author Contributions

HG and SH conceived of the case review, acquired, analyzed and interpreted the data, and drafted the article. AT conceived of the case review, acquired, analyzed and interpreted the data, and revised the article critically. MS, SK, HF, SB, PG, JG, and CN acquired, analyzed and interpreted the data, and revised the article critically. All authors approved of the final version of the manuscript and agreed to be accountable for all aspects of the work.

## Conflict of Interest

The authors declare that the research was conducted in the absence of any commercial or financial relationships that could be construed as a potential conflict of interest.

## Supplementary Material

Video S1 Angiography at CEPS level during balloon occlusion of the inferior vena cava.

Video S2 Selective angiography of the portal vein system.

## Abbreviations

CEPS: congenital extrahepatic portosystemic shunts
CT: computer tomography
HA: hepatic adenoma
HCC: hepatocellular cancer
HNF1-alpha: hepatic nuclear factor 1-alpha
HNF4: hepatic nuclear factor 4
FNH: focal nodular hyperplasia
L-FABP: liver fatty enzyme binding protein
MRI: magnetic resonance imaging
IVC: inferior vena cava
SV: splenic vein
SMV: superior mesenteric vein
PV: portal vein.
